# Multifunctional Magnetoelectric Sensing and Bending Actuator Response of Polymer-Based Hybrid Materials with Magnetic Ionic Liquids

**DOI:** 10.3390/nano13152186

**Published:** 2023-07-27

**Authors:** Liliana C. Fernandes, Daniela M. Correia, Mohammad Tariq, José M. S. S. Esperança, Pedro Martins, Senentxu Lanceros-Méndez

**Affiliations:** 1 Physics Centre of Minho and Porto Universities (CF-UM-UP), Universidade do Minho, 4710-057 Braga, Portugal; lilianafernandes1411@gmail.com (L.C.F.); pmartins@fisica.uminho.pt (P.M.); 2 Laboratory of Physics for Materials and Emergent Technologies, LapMET, Universidade do Minho, 4710-057 Braga, Portugal; 3 Centre of Chemistry, University of Minho, 4710-057 Braga, Portugal; 4 LAQV, REQUIMTE, Departamento de Química, Faculdade de Ciências e Tecnologia, Universidade Nova de Lisboa, 2829-516 Caparica, Portugal; tariq@fct.unl.pt (M.T.); jmesp@fct.unl.pt (J.M.S.S.E.); 5 IB-S Institute of Science and Innovation for Sustainability, Universidade do Minho, 4710-057 Braga, Portugal; 6 BCMaterials, Basque Centre for Materials and Applications, UPV/EHU Science Park, 48940 Leioa, Spain; 7 IKERBASQUE, Basque Foundation for Science, 48009 Bilbao, Spain

**Keywords:** hybrid materials, ionic liquids, smart materials, magnetoionic

## Abstract

With the evolution of the digital society, the demand for miniaturized multifunctional devices has been increasing, particularly for sensors and actuators. These technological translators allow successful interaction between the physical and digital worlds. In particular, the development of smart materials with magnetoelectric (ME) properties, capable of wirelessly generating electrical signals in response to external magnetic fields, represents a suitable approach for the development of magnetic field sensors and actuators due to their ME coupling, flexibility, robustness and easy fabrication, compatible with additive manufacturing technologies. This work demonstrates the suitability of magnetoelectric (ME) responsive materials based on the magnetic ionic liquid (MIL) 1-butyl-3-methylimidazolium tetrachloroferrate ([Bmim][FeCl4]) and the polymer poly(vinylidene fluoride-co-trifluoroethylene) (P(VDF-TrFE) for magnetic sensing and actuation device development. The developed sensor works in the AC magnetic field and has frequency-dependent sensitivity. The materials show voltage responses in the mV range, suitable for the development of magnetic field sensors with a highest sensitivity (s) of 76 mV·Oe^−1^. The high ME response (maximum ME voltage coefficient of 15 V·cm^−1^·Oe^−1^) and magnetic bending actuation (2.1 mm) capability are explained by the magnetoionic (MI) interaction and the morphology of the composites.

## 1. Introduction

In an era in which concepts such as the Internet of Things (IoT) and Industry 4.0 are key enabling technologies, miniaturized portable and multifunctional devices are becoming increasingly demanded. In this context, smart systems including sensors and actuators are essential components of the evolution of technology. However, geometrical and manufacturing complexity and the cost of commonly used materials and systems can be a drawback to their implementation [1,2].

Smart materials represent a suitable approach to develop a new generation of sensors and actuators to improve integration, cost efficiency and/or performance with respect to commonly used materials. In particular, polymer-based composites can be tailored with the ability to respond to external stimuli, including pH, temperature, stress and magnetic or electrical variations, among others [3,4]. This response is reproducible and of suitable magnitude and time-response to be used for sensors and/or actuators [5]. Among the various possible smarts materials, electroactive polymers (EAPs) have been gaining particular attention due to their light weight, mechanical flexibility, simple processing, compatibility with additive manufacturing technologies and tunability [6]. Thus, EAP-based smart materials have been applied in areas including sensors and actuators [4,7,8,9], biomedicine [10,11] and energy storage [12,13], among others [14,15].

In this scope, much attention has been paid to piezoelectric poly(vinylidene fluoride) (PVDF) and its co-polymers such as poly(vinylidene trifluoroethylene) (P(VDF-TrFE)). PVDF is a semi-crystalline polymer with a high dielectric constant, ionic conductivity, polarity and the largest electroactive response, including piezo-, pyro- and ferroelectricity, among polymers [16]. Due to their simple processability into different forms and shapes, PVDF and its copolymers have already been implemented in many different areas such as sensors, actuators and biomedicine [14]. The co-polymer P(VDF-TrFE) has the advantage of crystallizing in the ferroelectric β-phase when processed from solution or from the melt for specific TrFE contents [4].

Further, the combination of EAPs with ionic liquids (ILs) provides the opportunity to develop hybrid materials with improved and/or new functionalities such as electromechanical, mechanical, magnetic and electric properties considering specific target applications [15,17]. ILs are salts composed of anions and cations with a melting temperature typically below 100 °C [18]. Due to the many possible combinations of ions, ILs are known for their versatility and tunable properties; negligible vapor pressure; ionic conductivity; and high electrochemical, thermal, mechanical and chemical stability [19,20]. The presence of a paramagnetic element, typically a transition metal in the cation or anion, leads to the development of magnetic ionic liquids (MILs) exhibiting a permanent magnetic response when subjected to an external magnetic field [21]. The introduction of MILs into a polymeric matrix, such as solvent-casted P(VDF-TrFE), promotes the development of a new type of magnetoelectric (ME) material with magnetoionic (MI) coupling [9,22], where an electrical variation is induced by subjecting the materials to an external magnetic field [23]. Such an ME effect is related to the ionic movement of the cations and anions in the polymer matrix instead of magnetically induced dipolar variations [5].

Hybrid IL/polymer materials have been developed for implementation in different areas such as sensors and actuators, biomedicine and energy storage, but few studies concerning the combination of MILs with piezoelectric polymers with both MI and ME effects have been reported [17,24,25]. Additionally, and to the best of our knowledge, few studies concerning the use of piezoelectric MIL/polymer-based composites as magnetic sensors and actuators have been reported, in which the influence of the IL concentration or the cationic chain size of imidazolium-based ILs has been explored, but never the influence of the solvent evaporation temperature [5,9].

In this work, P(VDF-TrFE)/1-butyl-3-methylimidazolium tetrachloroferrate ([Bmim][FeCl_4_]) hybrid materials were developed via solvent casting, and their functional ME and bending actuation response was demonstrated.

## 2. Materials and Methods

### 2.1. Materials

[Bmim][FeCl_4_] (99%) was synthesized as reported in [26]. More specifically, 1-butyl-3-methylimidazolium chloride, [Bmim][Cl] (supplied by IOLITEC with a stated purity higher than 99%), was mixed in a 1:1 molar proportion with FeCl_3_·6H_2_O. Both compounds are solid at ambient temperature, so an ionic liquid aqueous solution was prepared before the addition of FeCl_3_ (available from Fluka with a stated purity over 99%). After synthesis, water was removed in a rotavapor. The final drying procedure consisted of vacuum-drying the sample for at least a day at moderate temperature (circa 330 K). N,N-dimethylformamide (DMF) (99.5%, Merck, Rahway, NJ, USA) and P(VDF-TrFE) (VDF-TRFE ratio of (70/30%) (350,000 g·mol^−1^, Solvay, Brussels, Belgium) were used as received. 

[Bmim][FeCl_4_] was selected based on its magnetic response and P(VDF-TrFE) based on its semicrystalline nature, high ionic conductivity and porous microstructure-forming capability [5].

### 2.2. Materials Processing

#### 2.2.1. Preparation of the Composite Films

**Figure 1:**
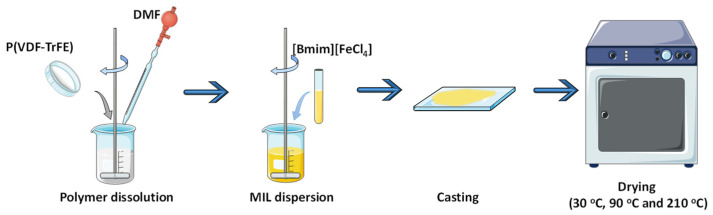
Schematic representation of the procedure used for the development of PVDF-TrFE and PVDF-TrFE/[Bmim][FeCl_4_] films.

P(VDF-TrFE) was dissolved in DMF at room temperature under mechanical agitation with a ratio of 15/85 weight percentage (wt.%). After complete polymer dissolution, [Bmim][FeCl_4_] (40 wt.%) was added to the solution. This concentration was selected based on [5], because of the higher value of magnetization. Hybrid films with a thickness of ~50 µm were obtained after spreading the solution on a clean glass substrate followed by the solvent evaporation in an oven (P-Selecta) at room temperature (~30 °C), 90 °C and 210 °C. The films thickness was 52.8 ± 5.0 µm, 158.2 ± 4.1 µm and 208.8 ± 22.2 µm with solvent evaporation temperatures of 210 °C, 90 °C and room temperature, respectively (Figure 1). Samples were prepared at different solvent evaporation temperatures, as this process strongly influences a sample’s morphology and functional response [15].

#### 2.2.2. Morphological and Functional Characterization

The morphology of the [Bmim][FeCl_4_]/P(VDF-TrFE) films was analyzed using a scanning electron microscope (SEM, NAnoSEM—FEI Nova 200, Hillsboro, OR, USA) with an accelerating voltage of 10 kV. The samples were previously coated with a thin gold layer using sputter coating (Polaron, model SC502, Quorum, Laughton, UK).

The ME effect was evaluated with Helmholtz coils powered with an AC current (Agilent 33220A signal generator, Keysight Technology, Santa Clara, CA, USA) to reach AC magnetic fields ranging from 0 to 2 Oe. The AC fields were applied along the thickness direction of the samples, and a Rigol DS1074Z (Rigol, Suzhou, China) oscilloscope was used to record the induced output voltage. Prior to the analysis, the samples were coated by 5 mm diameter gold electrodes, deposited on both sides of the samples via magnetron sputtering (Polaron SC502, Quorum, Laughton, UK). The determination of the transversal ME coefficient (*α*) was performed measuring the induced voltage using Equation (1), where the amplitude of the *AC* magnetic field is *H_AC_*, Δ*V* is the output voltage and d is the composite film thickness:

(1) $$\alpha =\frac{∆V}{H_{AC}\times d}$$

The actuator bending response of the materials was obtained with a high-definition Logitech HD 1080p Webcam camera, (Logitech, Lausanne, Switzerland) connected to a PC with 200 µm accuracy. The film actuator was clamped with two needles and submitted to the magnetic stimulation of a moving magnet (BX0C8-N52—K&J Magnetics, Pipersville, PA, USA—with a periodic movement from a maximum distance to the composite sample dmax = 2.2. mm to a minimum distance dmin = 0.1 mm, f = 0.1 Hz).

## 3. Results and Discussion

**Figure 2:**
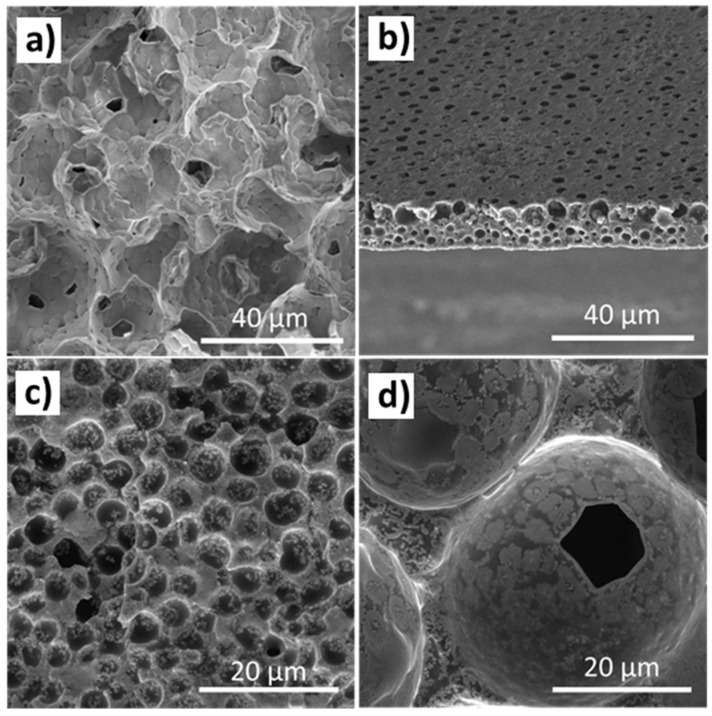
Representative SEM images of P(VDF-TrFE) and P(VDF-TrFE)/[Bmim][FeCl_4_] prepared with 40 wt.% content of MIL at different solvent evaporation temperatures: (**a**) room temperature P(VDF-TrFE); [Bmim][FeCl_4_]/P(VDF-TrFE) at (**b**) 210 °C, (**c**) 90 °C and (**d**) room temperature.

The morphology variations in the MIL/polymer materials with solvent evaporation temperature were evaluated via SEM (Figure 2).

Figure 2a shows the characteristic porous structure of electroactive P(VDF-TrFE) with an average pore size of 10.3 ± 2.2 µm [27]. Increasing the solvent evaporation temperature in pristine P(VDF-TrFE) leads to a more compact structure with the absence of pores due to the higher mobility of the polymer chains during solvent evaporation [28]. On the other hand, independently of the presence of the MIL and the solvent evaporation temperature used during the processing method, a porous structure is observed after the IL incorporation. This fact is an indication that the inclusion of the MIL in the polymer matrix induces porosity in the films, based on the strong interaction of the IL with the DMF solvent, the phase separation of the polymer and solvent phases and the solvent evaporation, with the free spaces left by the solvent occupied by the IL being dragged to the pores due to their interaction with the solvent [4]. Additionally, Figure 2 shows that the solvent evaporation temperature influences the pore size of the polymer matrix. As observed, the pore size decreases with increasing solvent evaporation temperature (Figure 2b–d) from a mean diameter of 2.2 ± 0.8 µm for solvent evaporation at 210 °C (Figure 2b) to 35 ± 6 µm for room-temperature-prepared materials (Figure 2d). Additionally, the pore size of the P(VDF-TrFE)/[Bmim][FeCl_4_] samples increase with decreasing solvent evaporation temperature, with the temperature influencing the solvent evaporation rate. Higher solvent evaporation temperatures lead to quick solvent removal, resulting in a higher number of spherulites with smaller porous radii during polymer crystallization. Further, increasing temperature leads to the polymer chains acquiring enough mobility to occupy the free spaces left by the solvent [28]. Contrarily, lower solvent evaporation rates lead to a phase separation process [27], and the low mobility of the polymer chains at low temperature does not allow them to occupy the free space left by the solvent, leading to a final microstructure with spherulites with small radii and higher porosity.

**Figure 3:**
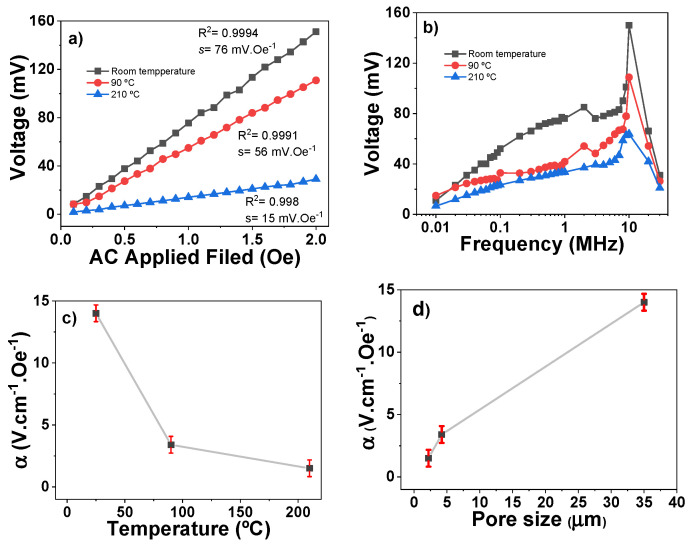
ME response of [Bmim][FeCl_4_]/P(VDF-TrFE) films: (**a**) ME voltage as a function of *H_AC_* intensity at 500 kHz; (**b**) ME voltage as a function of *H_AC_* frequency with *H_AC_* intensity at 2 Oe; (**c**) ME coefficient as a function of solvent evaporation temperature with *H_AC_* intensity of 2 Oe and frequency of 10 MHz and (**d**) ME coefficient as a function of the pore size with *H_AC_* intensity of 2 Oe and frequency of 10 MHz.

The effect of the solvent evaporation temperature on the ME sensing response of the [Bmim][FeCl_4_]/P(VDF-TrFE) composites with 40 wt.% of MIL is shown in Figure 3. Previous studies have shown the effect of different MIL contents in samples prepared at 210 °C [4,9].

Figure 3a shows a linear increase in the ME output voltage with increasing *H_AC_*, with fitting R^2^ > 0.998, characteristic of composites containing MILs composed with paramagnetic ions [29]. This effect is based in the interaction of the [FeCl_4_]^−^ anions with the applied *H_AC_*, where the anions move in the direction of the applied magnetic field. With a continuous variation in the direction of the magnetic field, the movement of the ions will generate the AC voltage signal in the electrodes of the material. Thus, the materials show voltage responses in the mV range, suitable for the development of magnetic field sensors with a highest sensitivity (*s*) of 76 mV·Oe^−1^.

As shown in Figure 3b, at lower frequencies, there is a high voltage increase with increasing frequency, while for higher frequencies, the opposite effect is observed. This fact is explained based on the dynamics and mobility of the ions within the pores of the polymer matrix. For lower frequencies, the ions have enough time to move within the pores, with this movement being maximized at ≈10 MHz. With increasing frequency, the ions’ mobility is reduced, and complete displacement does not occur, leading to ion relaxation processes and causing them to lag behind the fast excitation dynamics due to the increasing frequency [5,30].

The increase in the ME response with decreasing solvent evaporation temperature is shown in Figure 3c. As a consequence, the ME voltage coefficient *α* increases from 1.5 V·cm^−1^·Oe^−1^ to 14.1 V·cm^−1^·Oe^−1^ when the pore size increases from 2.2 μm to 35 μm (Figure 3d), which is explained by the reduced motion capability of the MILs in the smaller pores and the larger degree of confinement [31]. When such an ME response is compared to other P(VDF-TrFE)-based systems without magnetic ionic liquids, it is observed that it is 3 orders of magnitude higher than the value reported for P(VDF-TrFE)/CoFe_2_O_4_ nanocomposites (35 mV·cm^−1^·Oe^−1^) and in the same order of magnitude as the values reported for P(VDF-TrFE)/Vitrovac bi-layer laminates (66 V·cm^−1^·Oe^−1^) [32,33].

**Figure 4:**
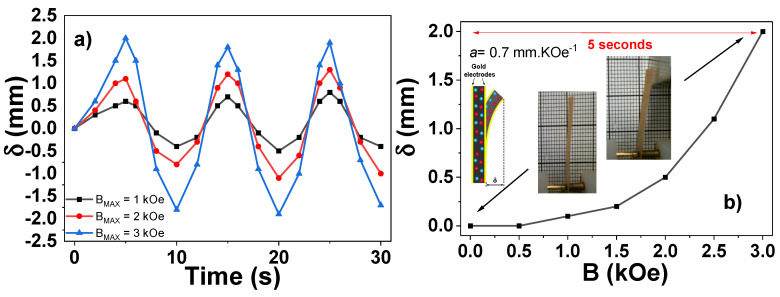
(**a**) Displacement and (**b**) bending response of the [Bmim][FeCl_4_]/P(VDF-TrFE) composite as a function of time (f = 0.1 Hz) and DC magnetic field intensity, respectively. As a scale reference, the size of the small squares in the images is 1 mm. The schematic inset represents the ions (cations and anions) movement into the polymer matrix upon the applied DC magnetic field.

Further, the magnetomechanical bending actuation response was evaluated in the hybrid materials, with the largest pore sizes and highest ME response observed for [Bmim][FeCl_4_]/P(VDF-TrFE) with 40 wt.% of MIL (Figure 4). 

Figure 4a,b show the displacement of the actuator tip with respect to the initial position and the bending response of the materials, respectively. It is also observed that the displacement, and consequently, the bending response of the sample increases with an increasing applied DC magnetic field. The maximum displacement (2.1 mm) is observed when the moving magnet is at the minimum distance to the sample (0.1 mm). In such a situation, the magnet applies to the [Bmim][FeCl_4_]/P(VDF-TrFE) sample an H_DC_ = 3 kOe. The periodic movement of the magnet also induces a periodic displacement of the samples, leading to bending of 0.2% and an actuation capability (*c*) of 0.7 mm·kOe^−1^. Thus, the highest bending response is achieved for a DC applied magnetic field of 3 kOe^−1^, with it being possible to tune the bending response for a specific application depending on the applied DC magnetic field. 

The response of the samples to the applied magnetic field is generated by the number and diffusion of the cations and anions near to the electrodes, as shown in the schematic representation of Figure 4b, with the bending response thus being governed by the mobility and ionic charge [15].

## 4. Conclusions

Hybrid films based on the magnetic ionic liquid [Bmim][FeCl_4_] and P(VDF-TrFE) with 40 wt.% filler content were prepared using the solvent casting technique at different solvent evaporation temperatures in order to tune samples’ morphologies. The morphologies of the P(VDF-TrFE)/[Bmim][FeCl_4_] composites depend on the solvent evaporation temperature, increasing the pore size with decreasing solvent evaporation temperature. The developed materials exhibit a double functional response: a magnetoelectric response of 14.1 V·cm^−1^·Oe^−1^ and a bending actuator response with a displacement of 2.1 mm and bending of 0.2%, which are maximized for the samples with the larger average pore size of 35 μm. Such results demonstrate the suitability of the MILs to be implemented in different polymeric matrices as innovative magnetic sensors and magnetically driven soft actuators, able to be prepared using additive manufacturing technologies.

## Data Availability

Not applicable.
